# Correction: An engineered biosynthetic–synthetic platform for production of halogenated indolmycin antibiotics

**DOI:** 10.1039/d1sc90180j

**Published:** 2021-09-03

**Authors:** Elesha R. Hoffarth, Sunnie Kong, Hai-Yan He, Katherine S. Ryan

**Affiliations:** Department of Chemistry, The University of British Columbia Vancouver Canada ksryan@chem.ubc.ca

## Abstract

Correction for ‘An engineered biosynthetic–synthetic platform for production of halogenated indolmycin antibiotics’ by Elesha R. Hoffarth *et al.*, *Chem. Sci.*, 2021, **12**, 8817–8821, DOI: 10.1039/D0SC05843B.

In the original manuscript, an inadvertent typo was made in the abstract. The authors employed a “tryptophan synthase gene” in this study, not a “tryptophanyl-tRNA synthetase gene,” as erroneously stated in the abstract.

The sentence beginning “To expand indolmycin…” should instead read “To expand indolmycin structural diversity, we introduce a promiscuous tryptophan synthase gene (*trpS*) into our *E. coli* production system and feed halogenated indoles to generate the corresponding indolmycenic acids, ultimately allowing us to access indolmycin derivatives through synthesis.”

The Royal Society of Chemistry apologises for these errors and any consequent inconvenience to authors and readers.

## Supplementary Material

